# The superior healing capacity of MRL tendons is minimally influenced by the systemic environment of the MRL mouse

**DOI:** 10.1038/s41598-023-42449-8

**Published:** 2023-10-11

**Authors:** Borys Frankewycz, Rebecca Bell, Monideepa Chatterjee, Nelly Andarawis-Puri

**Affiliations:** 1 Sibley School of Mechanical and Aerospace Engineering, Cornell University, Ithaca, NY USA; 2 University Hospital Regensburg, Regensburg, Germany; 3 Meinig School of Biomedical Engineering, Cornell University, Ithaca, NY USA; 4 Hospital for Special Surgery, New York, NY USA

**Keywords:** Trauma, Tendons, Animal disease models, Musculoskeletal models

## Abstract

Murphy Roths Large mice (MRL) exhibit improved tendon healing and are often described as a “super-healer” strain. The underlying mechanisms that drive the superior healing response of MRL remain a controversial subject. We utilized a tendon transplantation model between MRL and “normal-healer” B6-mice to differentiate between the contribution of MRL’s innate tendon and systemic environment to its improved healing capacity. Patellar tendons with a midsubstance punch injury were transplanted back into the same animal (autograft) or into an animal of the other strain (allograft). Findings at 4 weeks showed that the innate MRL tendon environment drives its improved healing capacity as demonstrated by improved stiffness and maximum load in MRL-grafts-in-B6-host-allografts compared to B6-autografts, and higher modulus in MRL-autografts compared to B6-graft-in-MRL-host-allografts. Groups with an MRL component showed an increase in pro-inflammatory cytokines in the 3 days after injury, suggesting an early enhanced inflammatory profile in MRL that ultimately resolves. A preserved range of motion of the knee joint in all MRL animals suggests a systemic “shielding effect” of MRL in regard to joint adhesiveness. Our findings 4-weeks post injury are consistent with previous studies showing tissue-driven improved healing and suggest that the systemic environment contributes to the overall healing process.

## Introduction

Tendon injuries are common clinical diseases with a rising incidence and a significant socio-economic impact^1–3^. Canonical tendon healing naturally results in scar tissue formation and fails to reconstitute pre-injury structure or mechanical properties^4,5^. Clinically, tendon scar tissue formation is often accompanied by dysfunction, pain or recurring injuries^6^. In contrast, “regeneration”, or scarless healing, is a healing process in which new growth completely restores portions of damaged tissue to their pre-injury state^7^. Accordingly, the path to achieving scarless tendon regeneration in adult mammals has prompted research into a wide range of approaches that include growth factors stimulation, biomechanical modulation, immunomodulation, cell therapy, mesenchymal stem cell and tendon progenitor cell research^4,8,9^. The scarcity of knowledge about the required physiological and biochemical mechanisms that would lead to a regenerative outcome has been a hurdle in the development of effective therapeutic methods for scarless tendon healing^10^.

The Murphy Roths Large (MRL/MpJ, (MRL)) mouse strain exhibits a superior healing capacity in multiple tissues including auricular cartilage^11,12^ myocardial tissue^13,14^, articular cartilage^15,16^, retina^17^, and cornea^18^, making it a powerful tool to unravel the mechanisms that drive effective adult mammalian healing. Recently, the improved healing capacity of this mouse has been shown to extend to tendon tissue^11,19–22^. Interestingly, some tissues in MRL mice, including skin lesions^23–25^, several tissues of the central nervous system^26^, and certain types of myocardial injuries^27^, do not exhibit its improved healing capacity. The tissue-specific healing potential of the MRL mouse, along with its unique systemic inflammatory environment has led to the generation of a myriad of hypotheses regarding the mechanistic drivers of its improved healing capacity. Ultimately, the mechanisms that drive the improved healing capacity of the MRL mouse are unknown.

Hypotheses that attribute MRL’s improved healing capacity to its unique systemic inflammatory environment^28–30^ are supported by evidence that suppressed inflammatory or systemic processes might allow a faster remodeling of the extracellular matrix (ECM) and ultimately a fully recovered architecture^14,31^. Other studies have found that compared to wild type mice, MRL mice exhibit differences in cell-related processes, such as an altered cell cycle, differences in cell cycle regulating proteins, enhanced proliferation, and blastema-mediated tissue restoration, creating cell-centered hypotheses as the drivers of its improved healing outcome^32–34^. Furthermore, the fact that the improved healing of MRL does not extend to every tissue, despite a shared systemic environment, has generated hypotheses centered around the biological milieu that is local to the tissue environment. For instance, the matrix generated by cultured MRL cells has been shown to promote an improved healing outcome in skin lesions^35^.

Our studies in tendons have led us to hypothesize that the innate tendon environment orchestrates its improved healing outcomes. This hypothesis is supported by multiple studies. First, we found that an improved repair outcome is achieved in sub-rupture fatigue damage injury in MRL mice; an injury that is characterized by a limited and muted systemic inflammatory response^22^. In addition, we found an uncorrelated capacity in the restoration of injured MRL ear and tendon midsubstance punch injuries, despite a shared systemic environment, suggesting that the local MRL tendon environment strongly impacts the healing outcome^30^. Furthermore, we showed that organ cultured MRL tendons retain their improved healing capacity despite complete isolation from the systemic environment^36^, supporting the role of the innate tendon properties as the main driver to improved healing. Lastly, we found that introducing hydrogel constructs consisting of MRL tendon-derived ECM into B6 patellar tendon defects led to an improved healing outcome, providing compelling evidence that the cues that drive the improved healing outcome of MRL tendons are sequestered within the MRL tendon matrix^37^. However, despite the capacity of organ cultured MRL tendons and MRL tendon-derived therapeutics to retain the improved healing capacity of MRL, the lack of full restoration of pre-injury structure and mechanical properties motivates evaluation of the contribution of the systemic environment to the healing response. Accordingly, a murine patellar tendon inter-strain transplantation model was developed^38^ to investigate the systemic influence on healing outcome. Cross-comparison of healing parameters of both MRL and B6 autografts and their corresponding allografts will allow a differentiation between the contributions of the systemic environment and the innate tendon environment. Based on our previous findings^30,37^, we hypothesize that the improved healing outcome of MRL tendons is driven by the innate tendon environment. Accordingly, we expect that (1) transplanted MRL tendon grafts will heal with improved structural and mechanical properties regardless of the mouse host utilized, and (2) the strain-specific systemic inflammatory response of the mouse host will not affect the quality of the healing tendon grafts.

## Results

### Graft length

**Figure 1:**
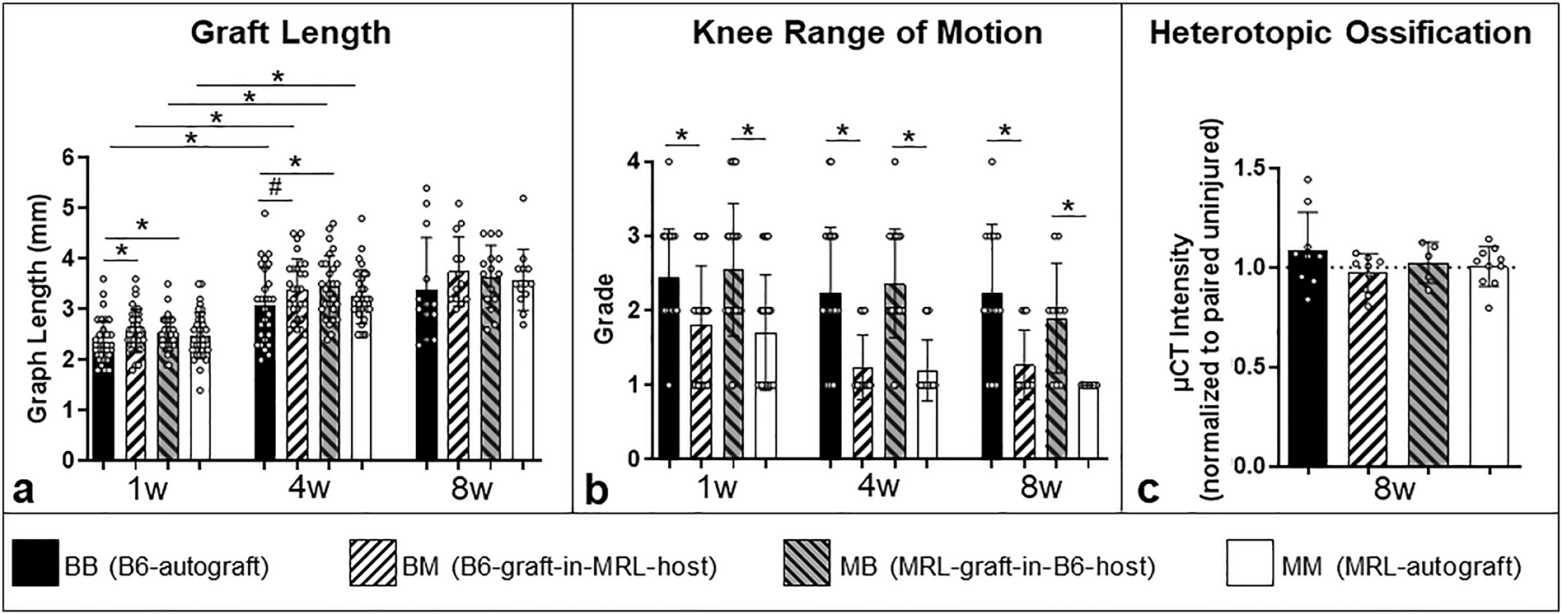
(**a**) Graft length: all groups had significantly higher graft length at 4 weeks compared to the pre-injury length. One week after surgery, B6-graft-in-MRL-host-allografts had a significant longer graft length compared to B6-autografts, however the differences were not observed at 4 or 8 weeks. (**b**) Knee range of motion: MRL mice had a significantly better ROM at all time points compared to B6 mice, regardless of graft source. Note that a low ROM grade corresponds to better movement and a higher ROM grade corresponds to a restricted movement. (**c**) Ossification: there were no differences in ossification between any of the transplant groups. (* indicates significance p ≤ 0.05, # indicates trend p ≤ 0.1, respectively).

Significant elongation of the grafts was observed in the first 4 weeks in all groups, likely reflecting a desirable strain due to muscular pull of the quad muscle on the graft through active knee movement. Differences in graft length between groups were detected between B6-autografts and B6-graft-in-MRL-host-allografts after 1 week with the latter being significantly longer than B6-autografts (Fig. 1a). In the second 4 weeks (week 4–8) there were no significant changes.

### Knee range of motion (ROM)

At all time points, passive knee range of motion was significantly better in all MRL animals, compared to B6 animals, regardless of the implanted graft (Fig. 1b).

### Ossification

No differences in average µCT intensity were found relative to uninjured contralateral in any transplant group at 8 weeks, suggesting limited ossification within the tendon in all groups as a result of the surgery (Fig. 1c).

### Mechanical testing

**Figure 2:**
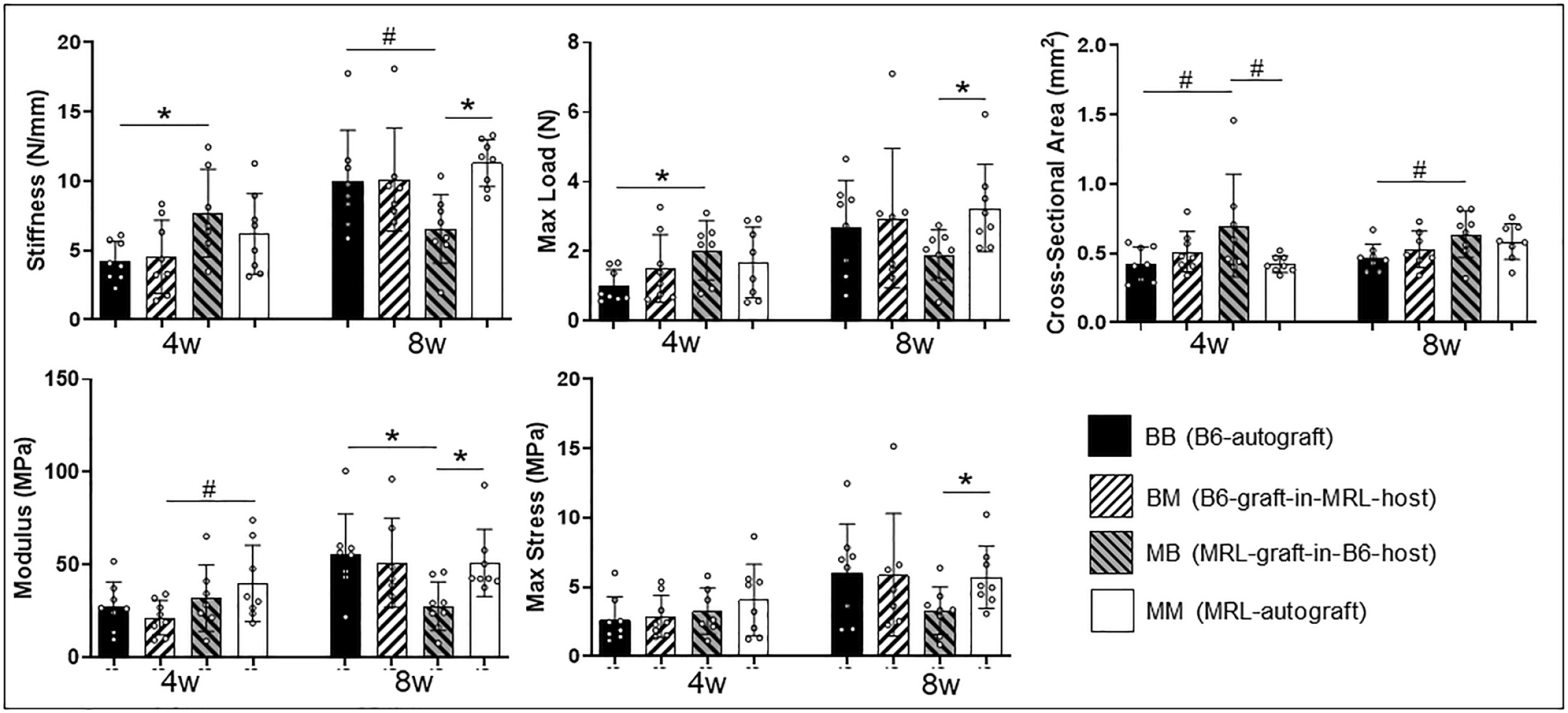
Stiffness and maximum load were increased in the MRL-graft-in-B6-host group compared to B6-autografts at 4 weeks and MRL-autograft group compared to the MRL-graft-in-B6-host group at 8 weeks. MRL-graft-in-B6-host-allografts had lower modulus compared to MRL- and B6-autograft groups and lower max stress compared to the MRL-autograft group at 8 weeks. (* indicates significance p ≤ 0.05 and # indicates trend p ≤ 0.1, respectively).

At 4 weeks, the MRL-graft in-B6-host group had significantly higher stiffness and maximum load than the B6-autograft group, supporting our hypothesis that MRL innate properties lead to improved healing (Fig. 2). At this time point, both autograft groups had smaller cross-sectional areas compared to the MRL-graft-in-B6-host group, and, in addition, the MRL-autograft group had higher modulus compared to the B6-graft-in-MRL-host group despite sharing a common MRL systemic environment (p ≤ 0.1). At 8 weeks, the MRL-autograft group had significantly higher stiffness, maximum load, modulus, and maximum stress compared to the MRL-graft-in-B6-host group, indicating that the nature of the healing environment that is associated with the autograft was ultimately more conducive to healing than that associated with an allograft. Accordingly, the MRL-graft-in-B6-host group had lower modulus (p ≤ 0.5) and stiffness (p ≤ 0.1) compared to the B6-autograft and MRL-autograft groups and significantly lower maximum load and maximum stress than the MRL-autograft group, suggesting that both autograft groups exhibited better healing than the MRL-graft-in-B6-host group at the 8 weeks.

### Histological assessment

**Figure 3:**
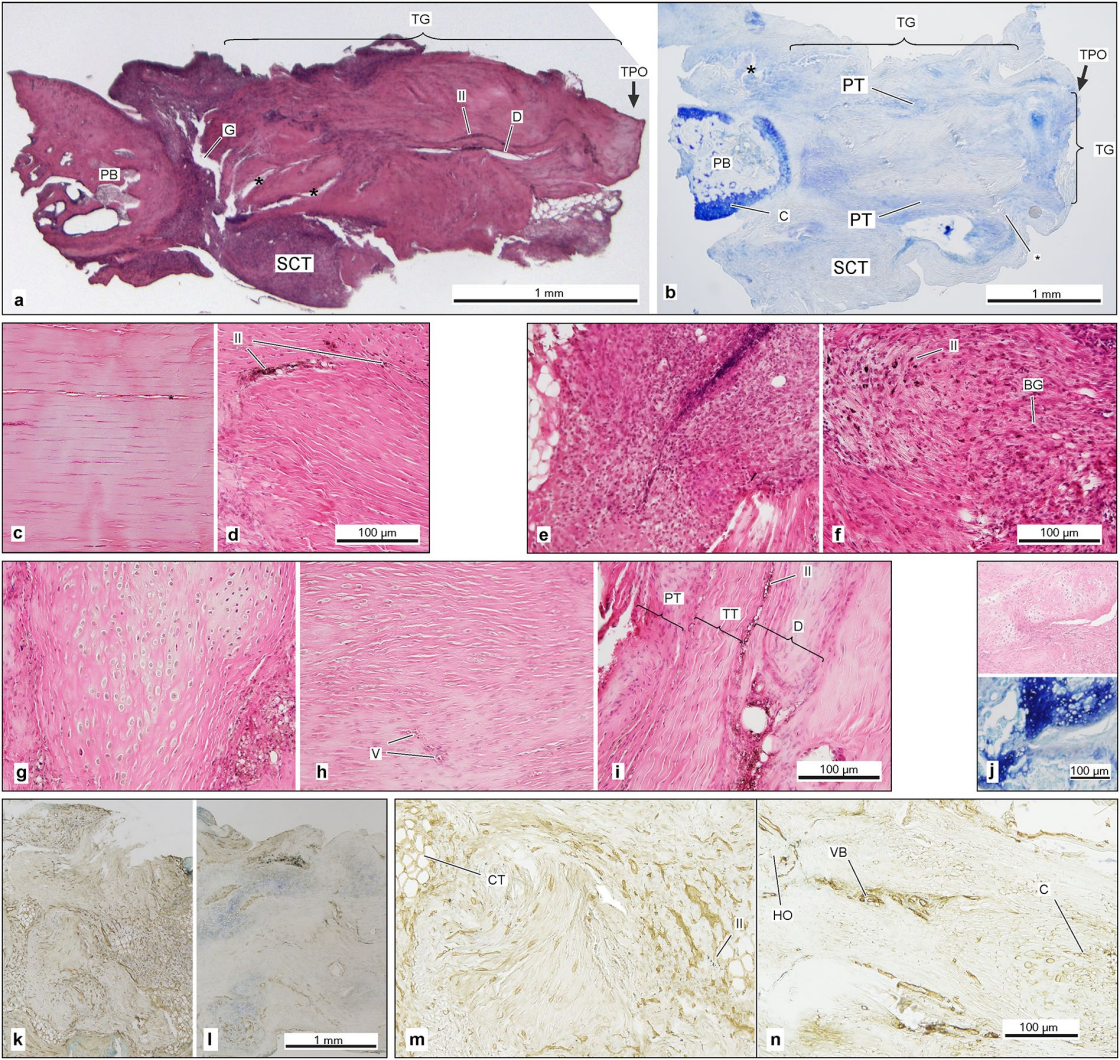
(**a**) H&E image (×1.25 magnification) of an extracted patellar-bone-graft-periostal-complex (1w, B6-graft-in-MRL-host-allograft): massive basophilic infiltration is found, especially in the peritendinous tissue (*PT*) and inside the graft close to the former central defect (*D*). India ink (*II*) is visible in low magnification, encircling the defect (*D*; *TG* tendon graft, *TPO* tibial periost, *PBR* patellar bone, *SCT* remnants of the surrounding soft connective tissue, * cutting artifact). (**b**) Corresponding Toluidine blue overview image (×4 magnification, 4w, B6-graft-in-MRL-host-allograft; *P* peritendineum, *C* periosteal cartilage of the patellar bone (*PB*)). (**c**) Healthy tendon with typical parallel-aligned fibrils and flat tenocytes and (**d**) transplanted tendon graft (**d**) with rather loose alignment of fibrils, and often oval/round fibroblasts (*II* = India ink marking the border of central defect with even rounder fibroblasts; H&E, ×20 magnification). High amounts of infiltrating cells found in both the peritendinous tissue (**e**) and within the defect area (**f**), particularly in B6-autografts at 1w. (*II* = dispersed India ink (black dots), indicating the proximity of the defect, *BG* = infiltrated basophilic granulocytes (blue dots)). (**g**) Representative MRL-autograft (4w) with altered cell morphology and mostly abundant fibril alignment within the proper tendon graft (**h**) Representative MRL-graft-in-B6-host-allograft showing a maintained tendon-typical parallel fiber alignment despite roundness of cells up to 8w (*V* = blood vessel). (**i**) Defect transition zone: the transition from native tendon tissue (*TT*) to defect-filling tissue (*D*) is recognizable by deprivation of native tendon-typical structure and cell morphology (B6-graft-in-MRL-host, 8w; *II* = India ink; *PT* paratenon). (**j**) Cartilage formation: rounded cells in H&E with circumjacent lacunae suggest cartilage formation, which is confirmed in the corresponding Toluidine-blue-stained section. (**k,l**) Representative slides (×4 magnification) of DAB-stained sections for collagen III at 1w (**k**) and 8w (**l**), with Touluidine blue counterstain. (**m**) Collagen III is mainly found in the early healing defect area (B6-autograft, 1w, ×10 magnification) and is still detectable at w8 (**n**) but is mostly concentrated in the increasingly present vascular bundles (*VB*), heterotopic ossification (*HO*) and around the newly formed cartilage (*C*; *II* India ink, *CT* surrounding loose connective tissue).

**Figure 4:**
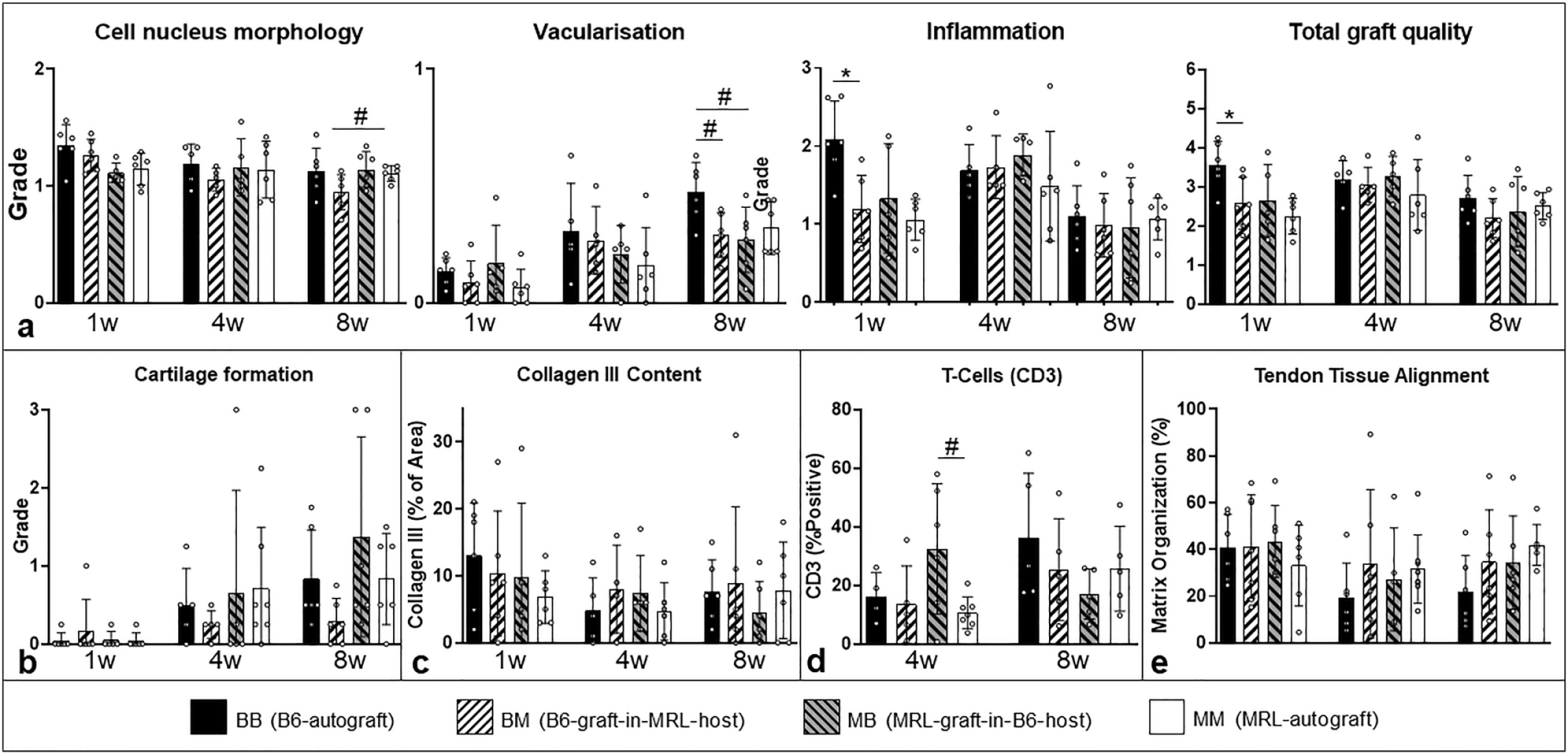
(**a**) Tendon quality: the ‘Total graft quality’ score is composed of the three separate scores ‘Cell nucleus morphology’, ‘Vascularization’, and ‘Inflammation’. At 1 week ‘Inflammation’ and ‘Total graft quality’ of B6-graft-in-MRL-host-allografts were improved compared to B6-autografts. At 8 weeks, inflammation decreased in all groups. Vascularization was highest in the 8 week B6-autografts (p ≤ 0.1 compared to both allograft groups; representative scoring images shown in Supplement 3, Fig. S-3a–c). (**b**) Cartilage formation did not differ between groups at any time point based on the histological grading score. (**c**) Collagen III did not differ between groups. (**d**) T-cells, indicated by CD3-positive staining, was higher in MRL-graft-in-B6-host-allografts compared to MRL-autografts at 4 weeks, but did not differ between groups at 8 weeks. (**e**) Alignment did not differ between groups at any time point. (* indicates significance p ≤ 0.05 and # indicates trend p ≤ 0.1, respectively).

Histological images (H&E and Toluidine blue) of all groups have shown vital tissue formation at all time points with various patterns of inflammation and tissue transformation. On H&E and Toluidine blue stained slides, the transplanted grafts were readily identifiable in low magnification images (1.25×) by their characteristic shape (Fig. 3a,b). Tendon microstructure with characteristic waved collagen fibrils was detected in almost all specimens, irrespective of study groups. After 1 week, the graft often appeared stretched and broad. Compared to native tendon, the tendon structure appeared loose, with partially disoriented alignment of the tendon fibrils (Fig. 3c,d). However, there were no significant quantified differences in the percentages of organized matrix fibers between study groups at any time point (Fig. 4e). Inflammation was observed in all groups, and was particularly prevalent in the peritendinous tissue in the first week (Fig. 3e,f).

Using our introduced histological scoring system (Table 1), the inflammatory response within the graft of B6-autograft mice at 1 week had the highest grade compared to the B6-graft-in-MRL-host group (BM) but subsequently decreased (Fig. 4a; representative scoring images shown in Supplement 3). In contrast, all animals with an MRL component exhibited lower initial inflammation at 1 week, which peaked at 4 weeks. Cell morphology within the grafts did not differ between groups. Cells mostly did not resemble the typical elongated tenocyte shape, particularly in the vicinity of the defect (Figs. 3g–i, 4a). As expected, collagen III was found in and around the injury defect of all groups (Fig. 3m–k) and remained high until week 8 (Fig. 3l,n) but did not differ between groups (Fig. 4c). In later stages the round cell nuclei occasionally were accompanied with surrounding lacunae, which suggested chondrocyte-like cell formation. Cartilage formation was confirmed in the corresponding Toluidine blue images (Fig. 3j). Cartilage formation was found in all groups with an increasing amount over time, but no significant differences were observed in the corresponding auto-/allograft comparisons (Fig. 4b). Vascularization steadily increased within the graft in all groups with different vessel diameters. At 8 weeks, vascularization was highest in the B6-autograft group, in particular compared to the both allograft groups (p ≤ 0.1), suggesting a potential suppressive effect of hypervascularization from MRL components (whether that be tendon-driven or systemic). In addition, B6-autograft mice had a significantly lower total score (worse outcome) compared to the B6-graft-in-MRL-host groups after 1 week, suggesting a positive early systemic influence from the MRL environment that most likely correlates to the decreased inflammatory response in the early healing phase. T-cells, whose presence may be indicative of graft rejection, were higher in the MRL-graft-in-B6-host group compared to the MRL-autograft group at 4 weeks (p ≤ 0.1) but not at 8 weeks (Fig. 4d; see Supplement 2 for quantification process).

**Table 1:** Histological scoring system of total graft quality and defect healing parameters adapted from work by Stoll et al.^66^.

	Points
Total graft quality score	
Cell nucleus morphology (CNM)	
Predominantly elongated, heterochromatic cell nuclei (tenocytes)	0
10–30% of the cells possess large, oval, euchromatic or polymorph heterochromatic nuclei	1
Predominantly larger, oval, euchromatic or polymorph, heterochromatic nuclei	2
Vascularization (V)	
Hypo-vascularized, like surrounding tendon (small or no capillaries)	0
Hyper-vascularized (increased numbers of small or larger capillaries)	1
Inflammation (I)	
No inflammatory cells present	0
< 25% cells in field of view are inflammatory	1
25–50% cells in field of view are inflammatory	2
> 50% cells in field of view are inflammatory	3
Cartilage formation	
Cartilage formation	
No cartilage formation	0
Little cartilage formation	1
Moderate cartilage formation	2
Significant cartilage formation	3
Defect healing parameters	
Defect cell infiltration (DCI)	
No cell infiltration	0
Some cell infiltration	1
Massive cell infiltration	2
Surrounding cell accumulation (SCA) around the former defect	
No additional cells around former defect	0
Slightly increased cell accumulation	1
Increased cell accumulation	2
Massively increased cell accumulation	3
Orientation of immediate surrounding tissue (OST)	
Tissue in parallel alignment at mid-length of defect	0
Tissue mostly in oblique alignment at mid-length of defect	1
Tissue mostly in perpendicular or random/indiscriminate alignment at mid-length of defect	2

A grade of 0 describes the most tendon-like phenotype with increasing values corresponding to increasing alterations in phenotype. Assignment of half points was permitted. Representative histological images for each grade shown in Supplement 3, Fig. S-3j–l.

### Histological defect healing parameters

**Figure 5:**
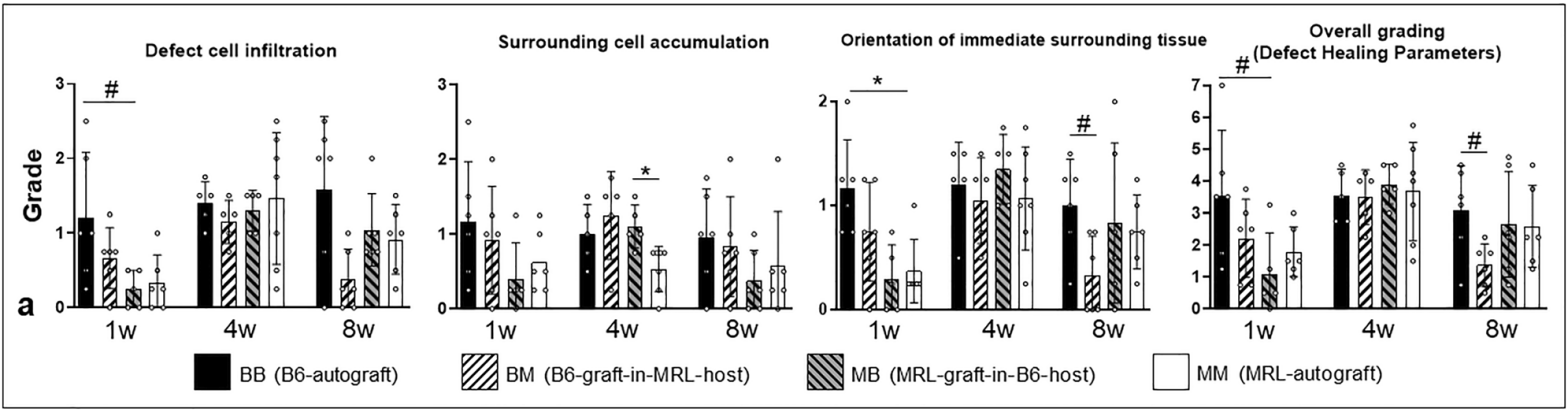
Histological defect healing parameters: in the overall grading (sum of the three sub-categories), MRL-graft-in-B6-host-allografts exhibited improved healing compared to the B6-autografts at 1 week and B6-graft-in-MRL-host-allografts exhibited improved healing compared to B6-auptgrafts at 8 weeks. (* indicates significance p ≤ 0.05 and # indicates trend p ≤ 0.1, respectively).

The MRL-graft-in-B6-host group exhibited a decrease in cellular infiltration (p ≤ 0.1) and a better orientation of the defect surrounding tissue compared to the B6-autograft group (p ≤ 0.5; see Fig. 5). The MRL-graft-in-B6-host group was improved compared to the B6-autograft group at 1 week (p ≤ 0.1) and the B6-graft-in-MRL-host group was improved compared to the B6-autograft group at 8 weeks as determined by the total grading of the central defect healing parameters (p ≤ 0.1; see Supplement 3, Fig. S-3j-l for representative histological grading images).

### Systemic cytokines

**Figure 6:**
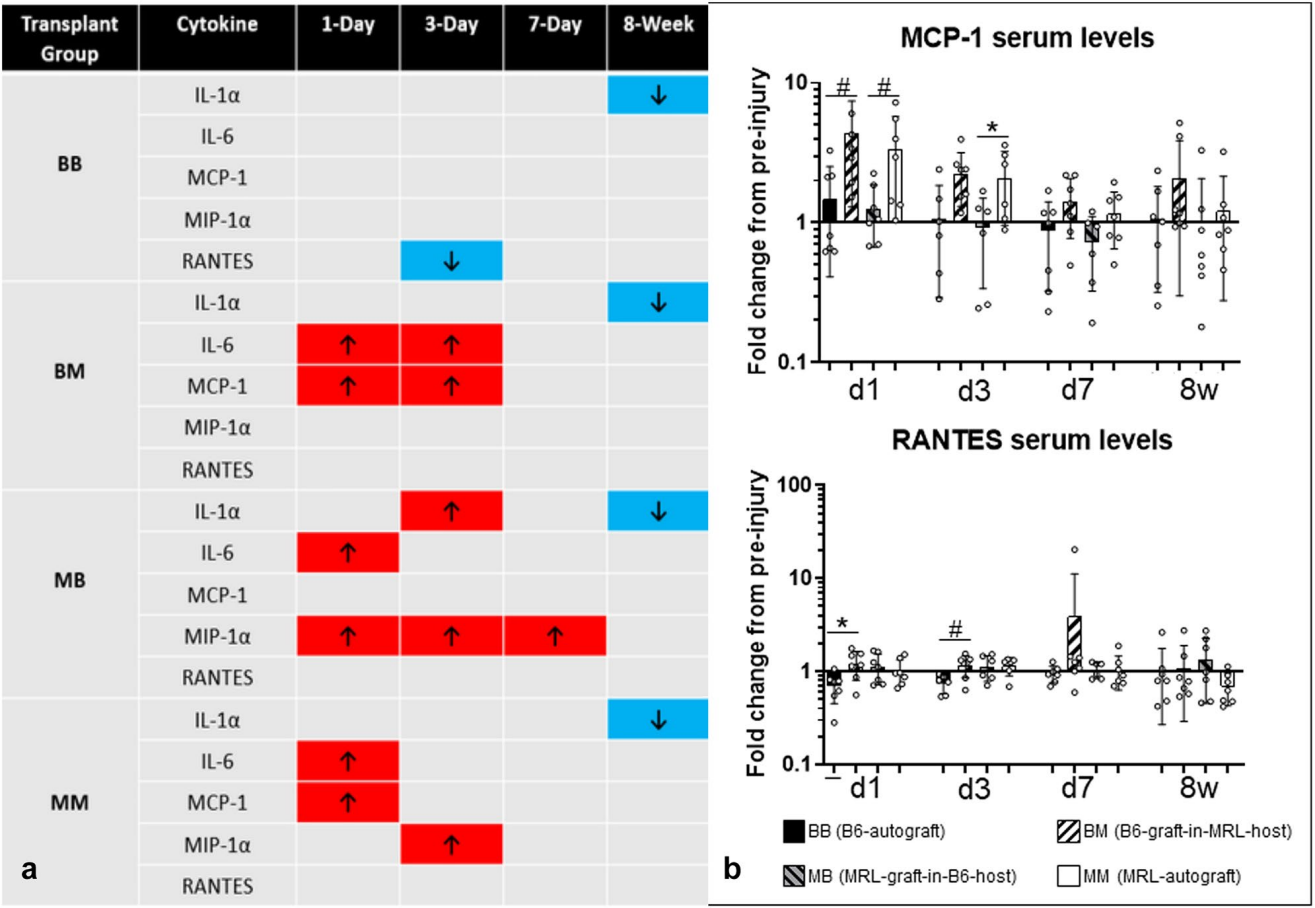
(**a**) Five blood serum cytokines were changed from pre-injury levels. Transplant groups with MRL components had increased pro-inflammatory cytokine levels (p ≤ 0.5). (**b**) Only MCP-1 and RANTES blood serum levels exhibited a difference from both their pre-injury levels and between transplant groups. Both cytokines were increased when the B6 tendon was placed in an MRL host relative to B6 host. (* indicates significance p ≤ 0.05 and # indicates trend p ≤ 0.1, respectively).

Out of the 14 blood serum cytokines quantified, IL-1a, IL-6, MCP-1, MIP-1a, and RANTES were further analyzed due to temporal differences from pre-injury in at least one transplant group (Fig. 6). Out of these 5 cytokines, only MCP-1 and RANTES exhibited differences between transplant groups at any time point. MCP-1 was higher in the B6-graft-in-MRL-host group than in the B6-autograft group at day 1 (p ≤ 0.1) and day 3 (p ≤ 0.05), and it was also higher in the MRL-autograft group than in the MRL-graft-in-B6-host group at day 1 (p ≤ 0.1). Similarly, RANTES levels were higher in the B6-graft-in-MRL-host group than in the B6-autograft group at day 1 (p ≤ 0.05) and day 3 (p ≤ 0.1). Interestingly, only transplant groups that had an MRL component (either graft or host) had an increase in pro-inflammatory cytokines within 3–7 days after injury. By 8 weeks post-transplant, the inflammatory response resolved, with IL-1a levels in all groups decreased relative to pre-injury, or not statistically different for the other cytokines. Baseline comparisons showed that only RANTES was significantly higher in the B6-autograft group compared to the MRL-autograft and B6-graft-in-MRL-host and B6-in-MRL-host groups (p ≤ 0.5, respectively). There was no baseline difference in the rest of the cytokines.

## Discussion

We have previously utilized organ culture and found that the superior healing capacity of MRL tendons is preserved despite the removal of the systemic environment^36^. In this study, we utilized our previously developed surgical tendon transplant model to determine the contribution of the systemic MRL mouse environment in driving its characteristic enhanced healing outcome since pre-injury level recovery in structure or mechanical properties was not achieved in organ culture^38^.

First, evaluation of clinical outcome using postoperative range of motion showed better mobility in all MRL-host groups, at all time points suggesting a systemic predisposition of MRL mice to maintain postoperative knee joint mobility. Clinically, joint movement after major knee surgery is largely inhibited by the swelling of the surrounding joint and soft tissues^39^. A few days after resorption of the swelling, formation of fibrotic tissue becomes the main inhibitor of joint movement^40^. Fibrosis, vascular proliferation, and synovial chondrometaplasia of the synovial tissue reduce lubricity and promote formation of adhesions within the joint capsule, potentially leading to arthrofibrosis^40,41^. The largely preserved knee mobility of the MRL animals with total restoration through week 8 shows a strong capacity of the MRL strain to impede fibrotic processes. This result is consistent with findings from Kallenbach et al. which showed significantly lower adhesion parameters accompanied by lower cell densities of the surrounding sheath tissue throughout an 8 week healing process in a partial defect model of MRL tendons in stark contrast to C57BL/6J mice^42^. However, since the inhibition of knee mobility is primarily a consequence of the adhesions of the joint capsule and the surrounding soft tissue complex, these results reflect different mechanisms than those associated with the healing capacity of the actual tendon tissue.

Our data supports that the local tendon environment drives the improved healing capacity of MRL tendons through the 4 weeks post injury. At 4 weeks, MRL tendons that healed in a B6 systemic environment had significantly higher stiffness and maximum load compared to the B6 autografts. Similarly, B6 tendons that healed in an MRL systemic environment did not exhibit an improvement in mechanical properties in comparison B6 autografts. Surprisingly, by 8 weeks, MRL tendons that healed in B6 hosts exhibited a deterioration in mechanical properties in comparison to MRL tendons that healed in an MRL environment. While it is not possible to definitively determine the cause for the deterioration of the MRL tendon allograft at 8 weeks, the typical time-course wherein the systemic environment is expected to contribute to the healing response is expected to be far earlier than 8 weeks. More specifically, healing follows three phases, namely *inflammation* (a few days), *proliferation* (up to 6–8 weeks), and the long-lasting *remodeling* phase (up to 2 years)^9,43^. During the first few days (*inflammatory phase*) fibroblasts, tendon stem/progenitor cells (TSPC), leucocytes, and other circulating cells and cytokines infiltrate the injury site and form a fibrin clot which serves as a temporary stabilizer and a groundwork for the subsequent tissue neo-formation^9^. The exposure to open blood vessels and the subsequent chemoattraction of systemic pro-inflammatory cytokines is inevitably most likely the biggest systemic influence during the whole course of tendon healing^44^, which cannot be avoided in an animal model. Interestingly, after 1 week, the central defect of MRL tendons showed an improved orientation of the immediate surrounding tissue when implanted into B6 compared to the B6 autograft, suggesting a dominant effect from the innate tendon on the healing outcome. This is in concordance with a study from Kajikawa et al., which showed that after 7 days the initially systemically circulation-derived mesenchymal cells are successively replaced by innate tendon-derived mesenchymal cells, which then participate in the ensuing proliferative phase^45^. In the *proliferation phase* collagen III is produced in high amounts by the integrated tenocytes, which is successively replaced by collagen I, resulting in a more fibrous tissue structure^9,46^. The biomechanical results at 4 weeks show significant differences between the B6-autograft and B6-graft-in-MRL-host groups, supporting the hypothesis of innate tendon influence on tendon healing at that stage. Interestingly, the biomechanical properties of MRL tendon allografts in B6 hosts decline by week 8 after surgery, during the *remodeling phase*. During this phase, the systemic environment is not a major driver as the predominant mechanism is the replacement of collagen III by collagen I through the function of tendon resident cells along with a general decrease in cell density^43,46^. Additionally, our data which showed that autografts generally had a higher modulus than their corresponding allografts by 8 weeks, at the time during which remodeling by the innate cellular environment occurs, is suggestive of a graft rejection. Consequently, our subsequent finding that T-cells were increased in the MRL tendons in the B6 systemic environment at 4 weeks could reflect the beginning of the rejection process that is manifested by 8 weeks.

The presence of key inflammatory cytokines in blood plasma was analyzed to further characterize the systemic response in MRL and B6 allografts and autografts. Other studies in MRL have shown a dampened inflammatory response to injury in comparison to C57Bl/6^30,47,48^. Interestingly, we found that groups with an MRL component (either host or graft) had elevated levels of pro-inflammatory cytokines in the first days after surgery. IL-6 is produced immediately and transiently in response to environmental stress, such as injury^49^ and MCP-1 and MIP-1a both recruit inflammatory cells to the site of injury^50,51^. Potentially, the early inflammatory period may be necessary to clear damaged tissue post-injury and instigate the healing cascade leading to the mechanical improvements in the MRL tissue^52^. Accordingly, the early increase in pro-inflammatory cytokines in groups with an MRL component might be critical to the improved healing outcome that is characteristic of MRL tendons^53,54^.

Several studies have investigated MRL tendon healing to identify the mechanisms that drive its improved healing capacity. Sereysky et al. showed that the improved healing capacity of MRL tendons extends to both, the biologically distinct acute midsubstance patellar tendon punch injury (wherein a robust systemic response is triggered), and sub-rupture fatigue tendon injury^55^. Since an improved healing outcome was found in sub-rupture fatigue injury, wherein the healing response is largely intrinsic to the tendon, data from this study supported the premise that the innate tissue’s local environment drives the superior healing response of MRL tendons. Paredes et al. reached a similar conclusion since they found no correlation between the level of healing in MRL ear and tendon injuries despite a similar shared systemic response^30^. Detection of an earlier increase in Hyaluronic acid and distinct TGF-β, PDFG, and bFGF contents in comparison to B6 mice insinuated that the composition of the provisional ECM during the proliferative phase could provide a bioactive structural template for a subsequent improved healing. This notion was further confirmed by an organ culture study which showed that midsubstance punch injured MRL tendons healed with improved stiffness and ultimate load compared to B6 with removal of all systemic contributions^36^. However, as the organ culture experiment effectively isolated the effect of the systemic environment from the local tendon environment, it did not isolate the effect of the tendon matrix from the cells that reside in the tendon. Characterization of MRL fibroblasts by Bedelbaeva et al. showed that they have an altered cell cycle with an arrest in the G2/M cell cycle phase that is linked to decreased p21 protein expression^32^. Interestingly, a p21^−/−^ knockout mouse exhibited a similar ear hole closure as MRL, suggesting that a prolonged G2/M phase extends cell proliferation until fully functional tissue is reestablished^32^. Increased cell proliferation with enhanced tissue healing was also found in the central nervous system^56^ and digit amputation^57^ of MRL mice, suggesting a cell-related proliferative blastema-mediated improved healing. Interestingly, the therapeutic effect of decellularized MRL provisional ECM was conserved when introduced in vivo to midsubstance punch injured B6 tendons supporting the notion that it is the MRL provisional ECM and not its resident cells that drive its therapeutic healing outcome^37^.

Conversely, studies in several tissues have shown a different immune response in MRL mice compared to normal-healer strains. Ear defect injury, which fully heals with a complete reformation of the cartilage plate^11,33^, triggers a muted inflammatory gene expression profile 24 h after injury compared to B6 mice that is accompanied by an increase in reparative genes^58^. Gourevitch et al. similarly found that most of the inflammatory related genes were downregulated, and only two were upregulated (compared to B6) at the very early phase (day 0) of healing of MRL ear cartilage defect^52^. This finding is in contradiction to our serological findings which showed an initial increase of pro-inflammatory markers. In contrast, MRL mice that were treated with an anti-inflammatory drug (meloxicam) had reduced levels of ear hole closure, demonstrating that some inflammatory processes are required for the scarless healing of the MRL mice^52^. In addition, a new kind of mast cells (MPO+/FCER1G−) was discovered as a potential contributor to scarless healing. Besides their distinct phenotype (lower levels of FCER1G, a common marker for mature mast cells) they have shown longer activity and higher migratory capacity. This cell type is expected to contribute to a more potent inflammation due to prolonged histamine release, which might inhibit formation of basement membrane and improve fibroblast migration^52^.

The synthesis of the unique and distinctive composition of the therapeutic provisional ECM of the tendon is influenced by both innate and systemic cofactors. In particular, growth factors, that are mainly involved in tendon healing cascades, are influenced by both innate and systemic processes. For example the expression of intrinsic TGFβ-1 in tenocytes, which is associated with fibrotic scar tissue formation, can be induced by IL-1β, which is produced by activated macrophages^59^. Interestingly, Kallenbach et al. discovered dichotomous roles of TGFβ-1 in B6 and MRL mice^42^. While TGFβ-1 triggered pro-inflammatory and ECM organization pathways in B6 tendons, it prompted transcription of different cell cycle regulatory genes, with negative enrichment of the senescence-related enzymes in MRL. Paredes et al. found differences in the downstream signaling pathways between the MRL and B6 strain despite no differences in TGFβ-1 content in the two strains. Similarly, Li et al. found that nearly half of the 21 genes increased (in ear defects of MRL but not in B6) are involved directly in wound repair, suggesting a much more complex molecular basis of wound repair of MRL^58^.

The surgical transplantation model presented a technical challenge and was likely the cause for the high variability in the data. An advantage of the transplant surgical model is that a mechanical load on the grafts, which is highly desired in order to stimulate tendon regeneration after injury^60^, is promoted. However, the highly invasive character of the surgery, particularly the double-sided tenotomy with concordant abolition of the endotendinous continuity and integrity makes the murine tendon susceptible for individual variations, making group-specific standardized reintegrations hard to identify. The invasive tendon extraction is coercively associated with an arthrotomy and synovial reaction which unavoidably unleashes an inflammatory reaction, swaying the outcome in favor of the systemic influence. In order to isolate the surgery-related systemic influence, less invasive models with fewer confounders (synovial interaction, individual joint mobility and weight bearing, etc.) like subcutaneous explanations into the animals` dorsal fascia might be favorable. However, such approaches can hardly provide the mechanical tension stimulus which is desired for tendinous healing. Moreover, the restricted range of motion of the B6 animals additionally might have compromised the full extent of the tensile stimulus, thus swaying the outcomes in favor of the systemic influence. However, it should be noted that as the surgical transplant model was used for all autograft and allograft groups in both mouse strains, we expect that this limitation does not introduce any bias in the results but ultimately suggests that only large effect sizes were detectable. Accordingly, it is likely that small but meaningful differences between groups were not detectable because of the surgical transplant model utilized. The autograft groups were deemed to be the optimal control groups to account for contributions from tissue surrounding the tendon. During tendon healing, cells are recruited from these tissues, such as the bursa, paratenon, and bone^61–63^. Less invasive models or sham groups would fail to account for the mouse strain-dependent contributions of the surrounding environment in our analysis and would essentially represent a completely different healing environment than the allograft groups.

While we assessed the mechanical properties of the tendon as the primary measure of the integrity of the repair, an assessment of the collagen I to collagen III ratio can also provide additional insight into the quality of the healing tissue. This analysis was not included in this study because of the limited number of slides that could be used for analysis since the thickness of the tendon was approximately only 0.1–0.2 mm. Despite these limitations, this study supports the hypothesis that the innate MRL tendon properties are driving the increased tendon healing properties. In the cross-strain transplantation model, the biomechanical and structural properties of MRL mice were almost retained, regardless of the host. As shown in previous studies, the systemic environment inevitably contributes to the healing process, in particular by distinct inflammatory cascades that unavoidably interact with cell and ECM processes. However, as demonstrated in studies isolated from the systemic environment, the MRL innate tendon properties hold the key components for improving tendon healing^36,37^. Therapeutically, this potentially could be used for enhancing healing processes of ruptured or damaged tendons by extracting and isolating these ECM components or through identification of their protein constituents. However, more studies are necessary in order to isolate the desired components and identify their key roles in the healing cascades. Future studies should also focus on the identification of MRL-specific cell properties that hold tenogenic potential^48,64^.

## Materials and methods

### Animal model and surgical procedure

**Figure 7:**
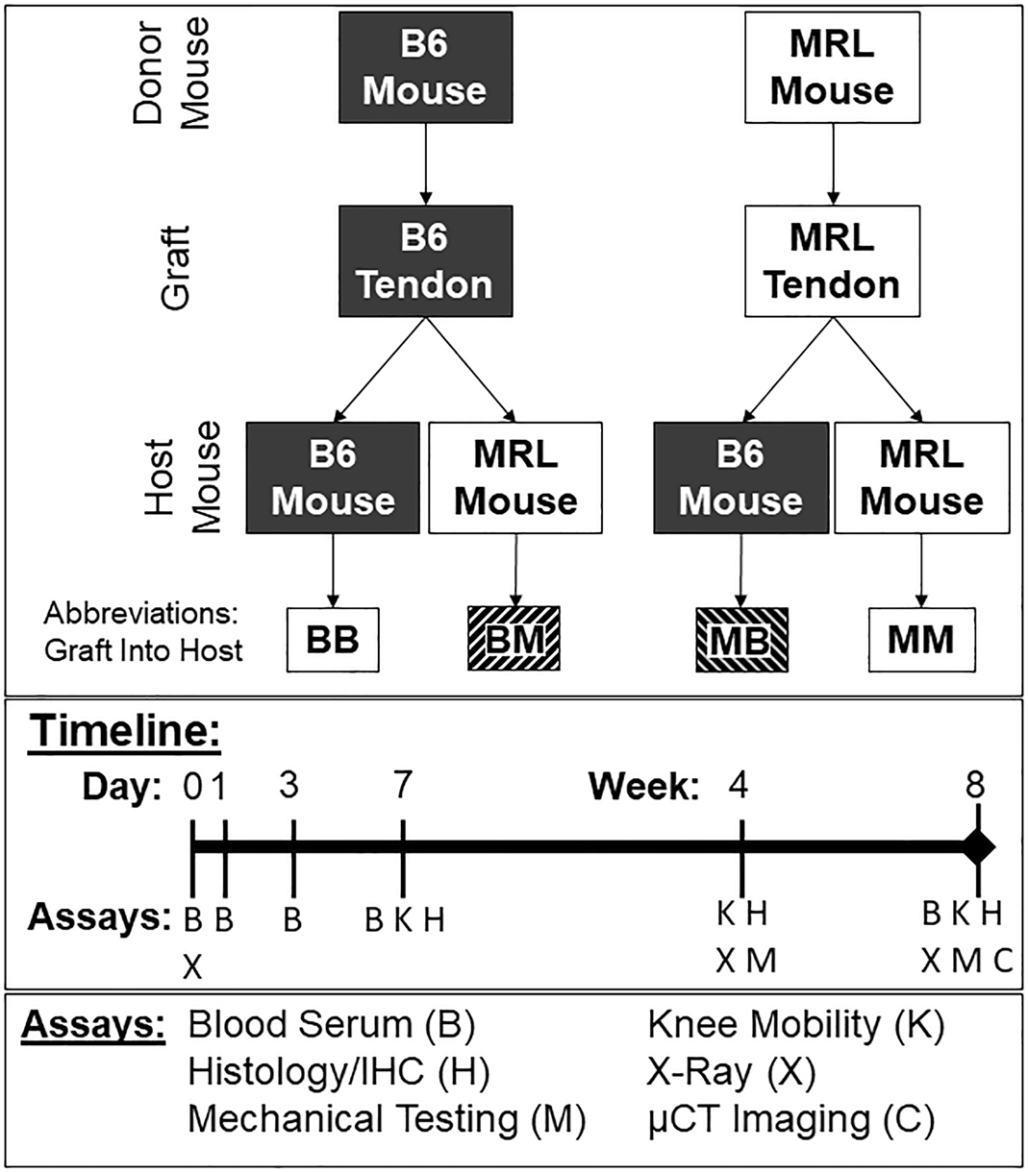
Schematic of experimental groups and study design. The four study groups are named with the first letter indicating the graft, the second indicating the host: BB (B6-autograft), MM (MRL-autograft), BM (B6-graft-in-MRL-host), and MB (MRL-graft-in-B6-host).

Following Cornell University’s Institutional Animal Care and Use Committee’s approval (protocol number 2015-0120 and appendant Refinement protocols), 15- to 16-week-old male B6 and MRL mice (n = 68, respectively) were purchased from The Jackson Laboratory (Bar Harbor, ME). All experiments were performed in accordance with the institutional guidelines and regulations, and all authors complied with the ARRIVE guidelines. Our previously established surgical technique was used to perform patellar tendon transplantations^38^. Briefly, after establishing inhalation anesthesia with isoflurane, the right patellar tendon was explanted, spread and flattened on a disinfected acrylic sheet. A 0.75 mm biopsy punch (Robbins Instruments, Chatham, NJ), coated with India ink for tracing the defect borders (Higgins, Chartpak, Leeds, MA), was used to create a central, full-thickness defect. The tendon was then re-implanted either as an autograft into the same animal or as an allograft into an animal from the other strain. Immediately after surgery, and at 4 and 8 weeks, sagittal X-ray images were acquired of the knee in a standardized 90° flexion in order to determine changes in graft length over time^38^. The four study groups were named with the first letter indicating the graft, the second indicating the host: BB (B6-autograft), MM (MRL-autograft), BM (B6-graft-in-MRL-host), and MB (MRL-graft-in-B6-host, Fig. 7). Animals were euthanized 1 week (early time point), 4 weeks (intermediate time point), and 8 weeks (late time point) after surgery for postmortem evaluation with CO_2_.

### Knee range of motion

To assess joint mobility, all animals (n = 33–34 per group) were examined to determine their maximum passive knee extension at 1, 4 and 8 weeks. Briefly, animals were anesthetized to ensure muscle relaxation, and their knee was manually extended from the natural flexed position into the most possible passive extension. The maximum possible extension was determined using a ROM grading scale with 5 grades in 15° steps (see Supplement 1). Scores were noted with grade 1 (normal/healthy extension) to grade 5 (highly inhibited extension). All assessments were performed by a trained staff member in a blind manner regarding the implanted graft.

### Histological and immunohistochemical (IHC) assessment

Histology and IHC was conducted (n = 5–6 per group per time point) postmortem at 1, 4, and 8 weeks (n = 5–6 per group per time point). The superficial soft tissue was removed and the patellar-tendon-patellar-bone-complex was harvested by undermining it with a backing and sharply transecting the quad muscle proximally and the tibial tuberosity distally from it. The tendon-bone-complex was placed on a plane custom-made cassette fitting made of Plaster of Paris and flattened to maintain the natural geometry of the tendon. A macro photograph was taken for subsequent orientation of the histologic images and determination of the tendon defect location (Nikon D5500 (Nikon Corp. Tokyo, Japan) mounted on an Amscope SM-4T-FRL microscope (United Scope LLC, Irvine, CA)). Specimens were fixed in zinc buffered formalin for 24 h, decalcified in formic acid (3 × 24 h), embedded into paraffin, and microtomed into 6 µm sections. Images were taken in 1.25×, 10× and 20× magnifications (Olympus DP74 microscope, Olympus Corp. Tokyo, Japan). Exploratory image analysis was conducted by two examiners on Hematoxylin–Eosin (H&E) and Toluidine blue stained sections (n = 2 per animal per staining). All subsequent grading was performed by two blinded independent examiners.

#### Total graft quality

Cell nucleus morphology (CNM), vascularization (V), and inflammation (I) were scored on H&E stained section images in 20× magnification using a scoring system adapted from work by Stoll et al.^66^ (representative scoring images in Supplement 3, Fig. S-3a–c). Cartilage formation within the whole graft was determined from Toluidine blue stained sections by grading the amount of blue stained areas on 20× magnified images^65^ (Table 1, representative scoring images shown in Supplement 3, Fig. S-3d–f). *IHC assessment:* Slides were DAB-stained for collagen III (Abcam ab7778, 1:500) or CD3 (Abcam ab135372, 1:1000) and counterstained with Toluidine blue. Images of the full width of the graft at the defect site were captured. The percentage of the stained collagen III area in the region of the injury was quantified using a custom MATLAB code (detailed description in Supplement 2). In order to identify a possible graft rejection at 8 weeks, T-cells, whose presence may be indicative of graft rejection, were identified and quantified. CD3 positive and negative cells were counted throughout the whole width of the tendon to account for the possibility that the response to injury includes the injured and non-injured areas. The percentage of total positive cells, positive cell density, and total cell density were calculated (representative histological images in Supplement 3, Fig. S-3g–i). Lastly, fiber alignment was assessed in the tendon proper from the Toluidine blue stained sections. Two blinded graders outlined all regions that exhibited matrix alignment in ImageJ while ensuring that surrounding fascia was excluded from analysis. The area of the aligned matrix was then divided by the total tendon area to determine the percent of the matrix that is aligned.

### Assessment of the injury site

The defect closure was evaluated on Toluidine blue stained sections by grading the following criteria (Table 1; representative histological scoring images in Supplement 3, Fig. S-3j–l): defect cell infiltration (DCI), surrounding cell accumulation around the former defect (SCA), and orientation of the immediate surrounding tissue (OST). The former hole defect was identified by overlaying the full-frame histology image over the macro photograph. The area with the surrounded India ink on the macro photograph was correspondingly identified visually on the histological slide (see Supplement 4 for detailed description) and defined as the former defect. Images that did not exhibit a clearly detectable former defect (India ink and exact recognizable defect location through macro photo overlay), or displayed the defect just partially were excluded from evaluation.

### Mechanical testing

Mechanical testing was conducted at 4 and 8 weeks (n = 7–8 per group per time point). Euthanized animals were frozen at − 20 °C and stored prior to testing. On the day of testing, animals were thawed at room temperature and digital images were captured of the in situ tendon to determine its width and thickness. The tendons were then extracted and gripped using custom-made sandwich grips. A preload of 0.1 N was applied for 2 min prior to pull-to-failure at 0.1%/s.

### Ossification

Prior to mechanical analysis, animals euthanized at 8 weeks underwent in situ micro-CT imaging while frozen (40 μm^3^/voxel) to evaluate heterotopic ossification in the graft (n = 7–8 per group). This new bone formation is indicated by high intensity signals within the soft tissue. The patellar tendon was manually segmented (Avizo) in sagittal sections by a blinded user and the average intensity of the tendon region was recorded. Due to the inability of the micro-CT machine to scan live animals, scans were only taken after euthanasia on frozen samples. The uninjured contralateral limb was used as a control.

### Systemic cytokines

Blood was drawn from all animals prior to surgery, and at 1, 3, 7 days (n = 33–34 per group per time point), and at 8 weeks (n = 14 per group) and stored in EDTA at − 80 °C. Systemic cytokines (IL-1α, -1β, -2, -3, -4, -6, -10, -12p70, -17, MCP-1, TNFα, MIP-1α, GCSF, and RANTES) were measured using a multiplex ELISA kit according to manufacturer’s instructions (Q-Plex™ Mouse Cytokine-Inflammation (14-plex), Quansys Biosciences, Logan, UT). Because systemic inflammation is known to occur rapidly after surgery and diminish within the first few days, as observed by day 3 in this study, intermediate blood samples were not analyzed to investigate the effect of systemic cytokines in this study.

### Statistics

Differences in graft length between groups was were assessed using Kruskal–Wallis Test with post hoc Dunn's Multiple Comparisons. A Mann Whitney test for unpaired observations was used for comparing BB vs. MB and BM vs. MM (highlighting the innate influence on scarless healing) and BB vs. BM and MB vs. MM (highlighting the systemic influence on scarless healing) with post hoc Bonferroni Correction at each time point, respectively. All data were expressed as mean ± standard deviation (SD). Significance was set at p ≤ 0.05 (denoted by ‘*’ on graphs) and trends were set at p ≤ 0.1 (denoted by ‘#’ on graphs).

### Supplementary Information

Supplementary Information.

## Data Availability

The datasets generated and analyzed during the current study are available from the corresponding author on reasonable request.
