# Supplementary material for: The superior healing capacity of MRL tendons is minimally influenced by the systemic environment of the MRL mouse
Source: Sci Rep. 2023 Oct 11;13:17242. doi: 10.1038/s41598-023-42449-8 (PMC10567747; doi:10.1038/s41598-023-42449-8)
Supplement: Supplementary file 1 — Supplementary Information. [file 41598_2023_42449_MOESM1_ESM.pdf]

Supplement 1: Grading of Knee Range of Motion (ROM)

Passive knee ROM, strictly speaking passive knee extension, was determined by laying the anesthetized mouse on the right lateral position with the right hind limb on an underlying range scale (Fig. S-1), manually fixating the femur onto the corresponding position on the scale. The knee was passively extended by pulling the shank into extension until a mechanical resistance occurred. The scale was divided into 15° steps with grade 1 (normal/healthy extension) to grade 5 (highly inhibited extension). All animals exhibited normal extension (Grade 1) prior to surgery.

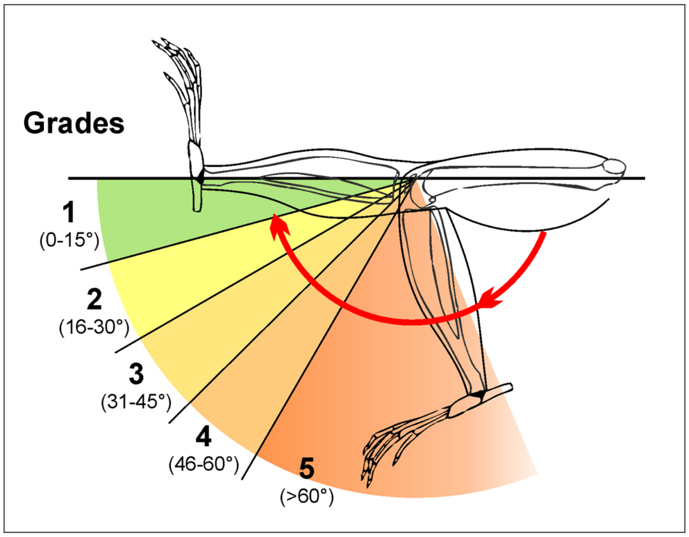

Fig. S-1: Range scale with degrees of knee ROM by manual passive extension in anesthesia.

Supplement 2: Quantification of collagen III using a custom MATLAB code

2.1. Image processing

For collagen III quantification, the central width of the transplant area of DAB stained slides for collagen III was used (n=5-6, 2 slides per animal; Fig. S-2a). Using a 10x magnification, three consecutive full-width images were taken using the same microscope settings (Fig. S-2b). The surrounding soft tissue was manually deleted (Fig. S-2c+d, ImageJ, Ver. 1.51n, NIH, Bethesda, MD). Using a custom MATLAB code (supplement 2.2), all brown pixels were identified with a colorThresholder using predefined HSV (*hue, saturation, (brightness) value*) values, generating a binary pixel mask (Fig. S-2e). Collagen III was quantified by dividing the amount of brown pixels by the total pixel amount, which was similarly counted without the brown color threshold (supplement 2.3). The HSV threshold values for DAB where previously validated by two separate investigators.

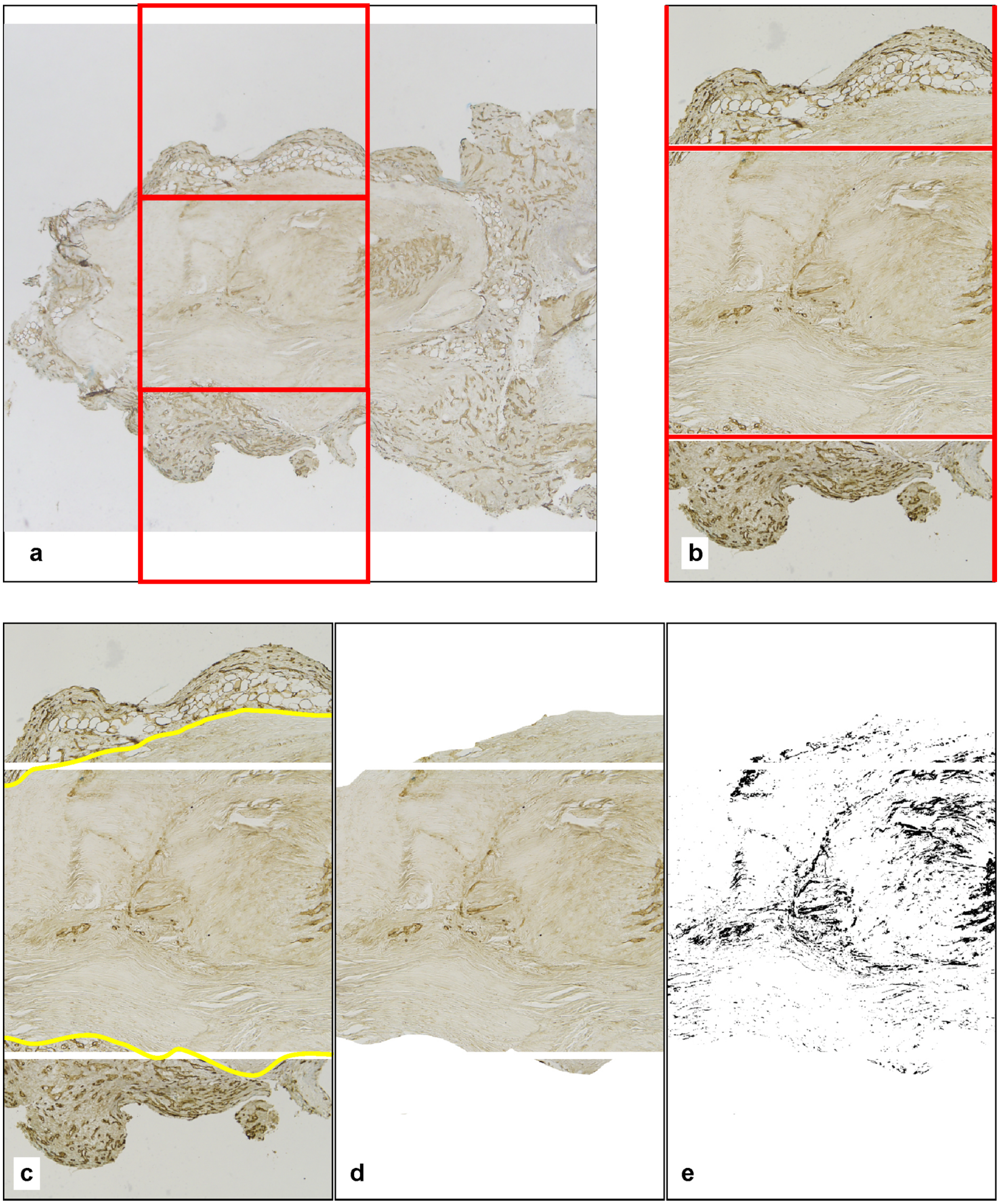

Fig. S-2: Image processing for quantification of collagen III

2.2. Custom MATLAB Codes for DAB Threshold

```
function [BW,maskedRGBImage] = createMask(RGB)
%createMask  Threshold RGB image using auto-generated code from colorThresholder app.
%  [BW,MASKEDRGBIMAGE] = createMask(RGB) thresholds image RGB using
%  auto-generated code from the colorThresholder app. The colorspace and
%  range for each channel of the colorspace were set within the app. The
%  segmentation mask is returned in BW, and a composite of the mask and
%  original RGB images is returned in maskedRGBImage.

% Auto-generated by colorThresholder app on 10-Aug-2018
%-------------------------------------------------------

%H, S, and V values optained from Borys' specifications for brown
Hmin=6; Hmax=43;

Smin=21; Smax=254;

Vmin=95; Vmax=233;
% Convert RGB image to chosen color space
I = rgb2hsv(RGB);

% Define thresholds for channel 1 based on histogram settings
channel1Min = Hmin/256;
channel1Max = Hmax/256;

% Define thresholds for channel 2 based on histogram settings
channel2Min = Smin/256;
channel2Max = Smax/256;

% Define thresholds for channel 3 based on histogram settings
channel3Min = Vmin/256;
channel3Max = Vmax/256;

% Create mask based on chosen histogram thresholds
sliderBW = (I(:,:,1) >= channel1Min ) & (I(:,:,1) <= channel1Max) & ...
    (I(:,:,2) >= channel2Min ) & (I(:,:,2) <= channel2Max) & ...
    (I(:,:,3) >= channel3Min ) & (I(:,:,3) <= channel3Max);
BW = sliderBW;

% Invert mask
% BW = ~BW;

% Initialize output masked image based on input image.
maskedRGBImage = RGB;

% Set background pixels where BW is false to zero.
maskedRGBImage(repmat(~BW,[1 1 3])) = 0;

end
```

2.3. Custom MATLAB Codes for quantification of the DAB / whole area ratio

```
%input multiple images from one folder

prompt = {'Enter Sample ID'};
dlg_title = 'Sample ID';
num_lines= 1;
answer = inputdlg(prompt,dlg_title,num_lines);
sampleID = str2num(answer{1});
Hfig = figure('Tag', 'Show_Figure');
set(Hfig,'FileName',fullfile(pwd,'*.tif')); % Change this if you want .jpg
ofile = get(Hfig,'FileName');

[ofnm, pth] = uigetfile(ofile, 'Please Select Images');
if(ofnm==0)
disp('Cancelled ..');
return;
end
[pth,fname,ext]=fileparts(fullfile(pth,ofnm));
Din = dir(fullfile(pth,['*',ext]));
Ns = length(Din);
sr_v = listdlg(...
'PromptString', 'Choose Files', ...
'SelectionMode', 'Multiple', ...
'Name', 'Conversion File List', ...
'InitialValue', [1:Ns], ...
'ListString', {Din.name});
if(sr_v==0)
disp('Canceling ...');
return;
end
close all
num=length(sr_v); % Calculate number of images

row_header = [];
```

```
warning('off','all');
% Suppresses warnings that images are too large to view and will be
% displayed at 67%

% number_of_files = number_of_files-1;

%set headers for output data
data= cell(num+1,3);
data{1,1}= 'File Names';
data{1,2}= 'Number of Brown Pixels';
data{1,3}= 'Number of Total Area';

%makes a folder for the bwn pics
mkdir(answer{1});

%read file
 for q=1:num %length(dir)
       try
        %Read File
        fname = char(Din(sr_v(q)).name);
        file=strcat(pth,'/' ,fname);
        imrbg=imread(file);

     %convert image to grayscale
    imgray=rgb2gray(imrbg);

    %%Threshold the grayscale image
    % T=.997 is 1/256, passes everything except pure white
   T=.997;
    imbw= imbinarize(imgray, T);
    imbw= ~imbw;

    %use BrownFilter to threshold image for brown
    [imBwnThresh, maskedRGBImage] = BrownFilter(imrbg);

    %remove small pixel groups from bwn image
    imBwn2= bwareaopen(imBwnThresh,6);

% %     makes a file for the bwn images and puts file in folder
    picName=strcat('Bwn ', fname);
    imwrite(~imBwn2, picName);
    movefile(picName, answer{1});

% % display images
%     figure(), imshow(file), title('original image');
% %     figure, imshow(imgray), title('grayscale image');
%      figure, imshow(~imbw), title('b&w thresholded image');
% %       figure, imshow(~imBwnThresh), title('Thresholded for Brown');
%       figure, imshow(~imBwn2), title('Bwn with small removed');

%%calculate amount of brown and total area by summing the number of white pixels
    BwnPix= 0;
    tendonPix= 0;

    for a=1:size(imbw,1)
        for b=1:size(imbw,2)
            %white pixels are tendon but are displayed as black
            tendonPix= tendonPix+ imbw(a,b);

      %brown tendon parts are thresholded as white but displayed as black
            BwnPix= BwnPix+ imBwn2(a,b);
        end
    end

    BwnPerc= BwnPix/tendonPix;

    %store results in data cell
    data{q+1,1} = fname;
    data{q+1,2}= BwnPix;
    data{q+1,3} =tendonPix;
           end
     end
```

Supplement 3: Representative images for the histological scoring systems of the Total Graft Quality, Cartilage Formation, CD3 cells and Defect Healing Parameters

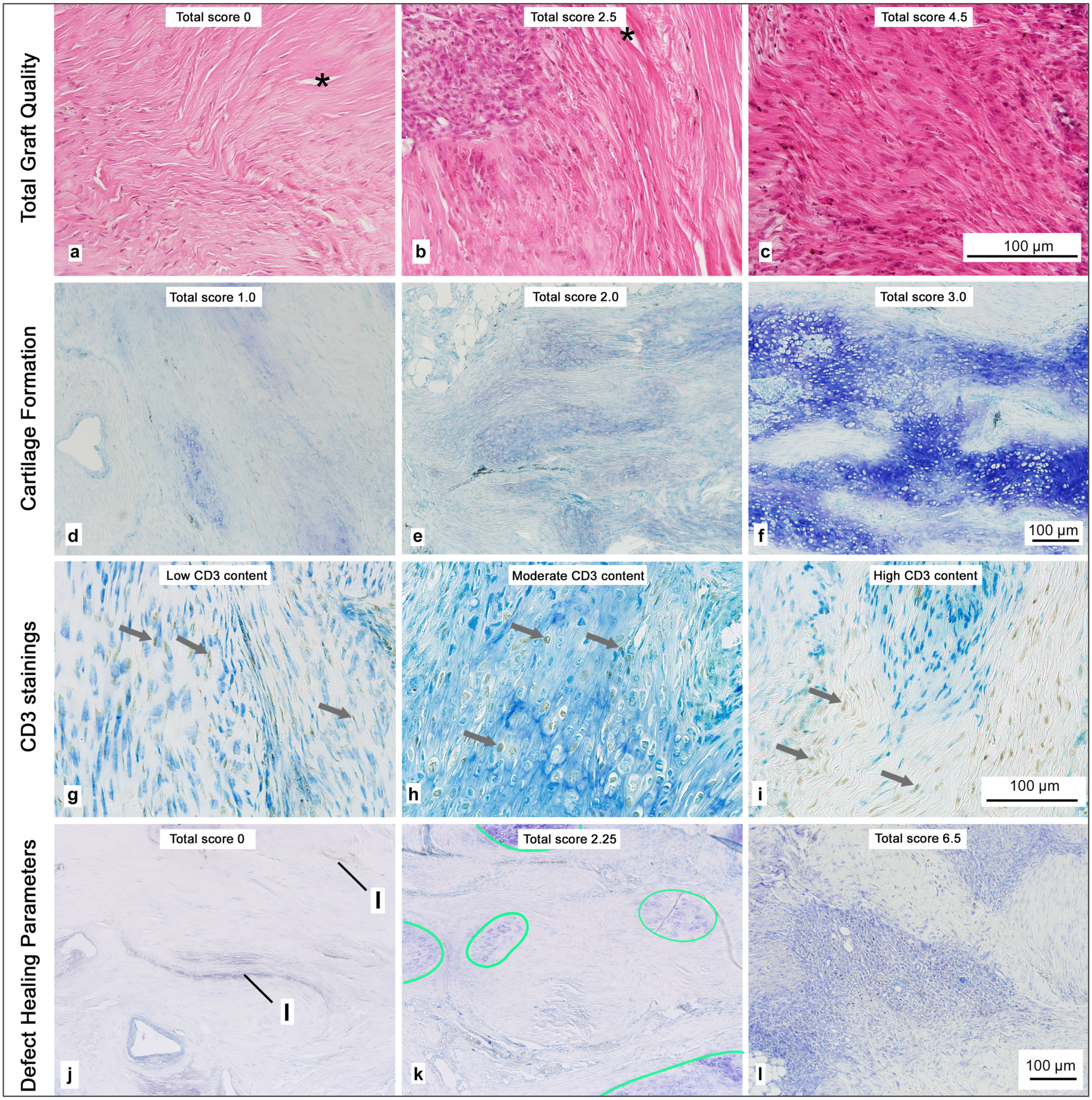

Fig. S-3(a-c): Representative images used for grading of the Total Graft Quality (H&E, 20x magnification): **(a)** Graft shows predominantly elongated, heterochromatic cell nuclei (tenocytes), with no signs of inflammation and vascularization. **(b)** A representative image showing 10-30% of the tendon cells as possessing a large, oval, euchromatic or polymorphic heterochromatic shape (CNM=1). The left portion of the image shows a locally confined basophilic (purple) inflammatory response (I=1.5) without increased vascularization (V=0). **(c)** A representative image showing predominantly large and oval

nuclei (CNM=2), and 25-50% inflammatory cells (I=2) in the field of view, with some increased vascularization (V=0.5). C = capillary, * = cutting artifacts.

(d-f) Representative images of the grading of Cartilage formation (Toluidine blue, 10x magnification) with **(d)** little, **(e)** moderate, or **(f)** high cartilage formation.

(g-i) Representative images of CD3+ cell counting (DAB-stain for CD3 with Toluidine blue counterstain, 20x magnification). Cells were counted manually using ImageJ Cell Counter plugin (Ver. 1.51n, NIH, Bethesda, MD) with n=5 per group, 2 slides per animal. **(g)** shows a low (~10%), **(h)** a moderate (~20%), and **(i)** a high percentage (40%) of CD3 positive cells.

(j-l) Representative images used for grading of the Defect Healing Parameters (Toluidine blue, 10x magnification). **(b)** The defect in this sample is closed without cell infiltration (DCI=0) or additive cell accumulation (SCA=0), and with a parallel aligned tissue at the mid-length of the former defect (OST=0); I = India ink marks the borders of the former punch defect. **(c)** This sample shows some increased cell infiltration (DCI=1), slightly increased cell accumulation (SCA=1) and some oblique alignment at mid-length of defect (OST=0.25). Additionally, some cartilage formation is found (green line). **(d)** In comparison to b and c this sample shows massive cell infiltration (DCI=2) with highly increased surrounding cell accumulation (SCA=2.5) and mostly randomly aligned surrounding tissue (OST=2).

Supplement 4: Definition of center defect area

The identification of the former defect area was performed utilizing the corresponding macro photographs of the explanted grafts (see Fig. S-1).

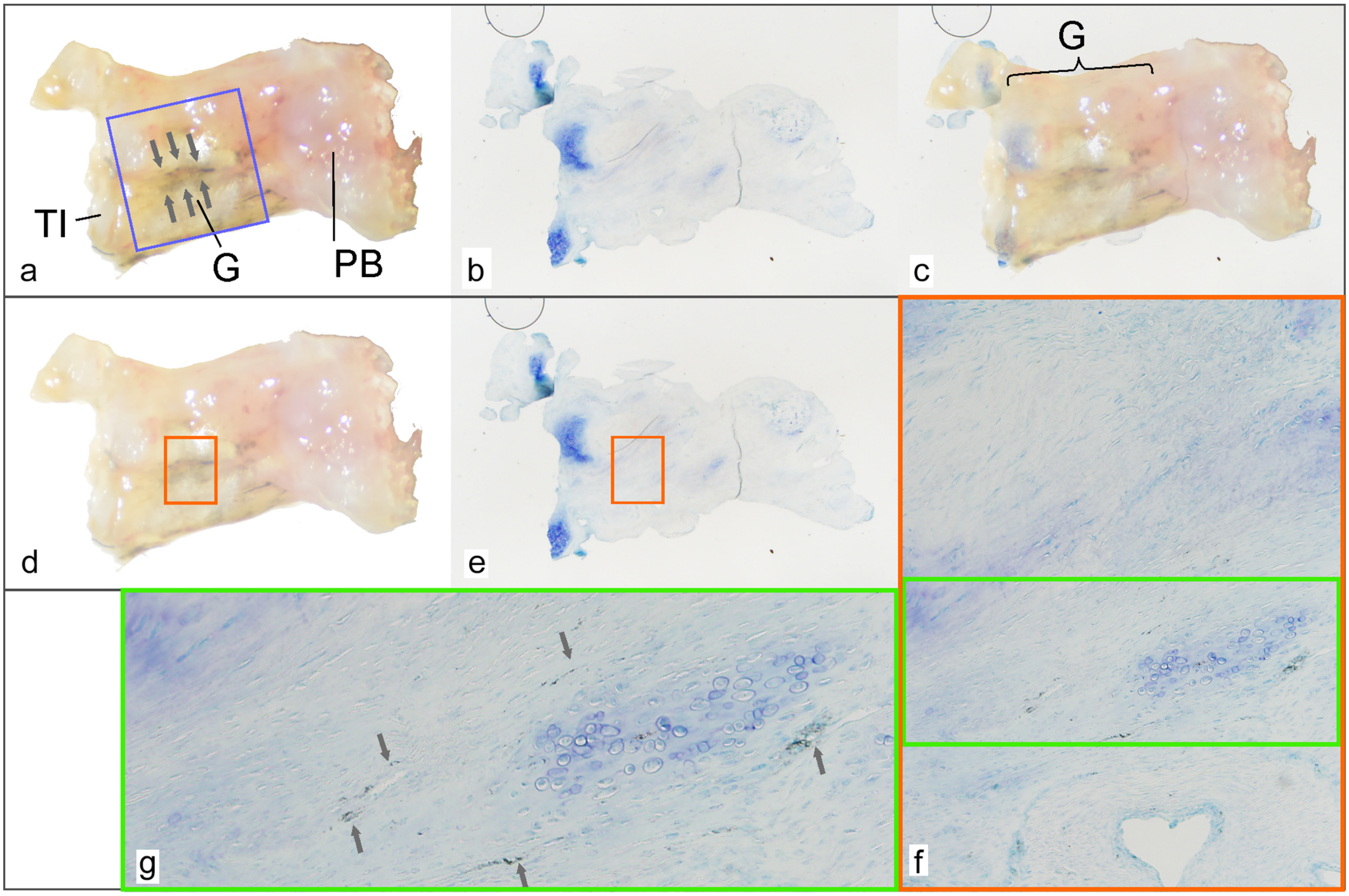

Fig. S-4: After sacrifice, the whole patellar bone (PB) – graft (G) – tibial insertion (TI) -complex **(a)**, was isolated by sharply transecting the quad muscle at the proximal side and sharp scraping of the a periost at the tibial tuberosity at the distal side of the graft. In the area of interest (graft in blue square) the macroscopically visible India ink markings were used to identify the original defect (grey arrows). The macro photograph and the corresponding 1.25x magnified histology image **(b)** was digitally overlaid, allowing an exact recognition of the corresponding anatomical parts **(c)**. Accordingly, the area of interest (orange frame in **d**), was identified on the histology slides (**e**), where the 10x magnified images where taken (**f**). (**g**) shows an enlarged image of the defect area (green frame). Here, the correspondingly visible India ink was used to identify the center defect boundaries (grey arrows) for evaluation of the Defect Healing Parameters.
